# Evaluation of Gait Pattern and Lower Extremity Kinematics of Children with Morquio Syndrome (MPS IV)

**DOI:** 10.3390/diagnostics11081350

**Published:** 2021-07-27

**Authors:** Jose J. Salazar-Torres, Chris Church, Thomas Shields, M. Wade Shrader, Lydia Fisher, William G. Mackenzie, W. G. Stuart Mackenzie

**Affiliations:** Nemours A.I. duPont Hospital for Children, 1600 Rockland Rd., Wilmington, DE 19803, USA; chris.church@nemours.org (C.C.); thomas.shields@nemours.org (T.S.); wade.shrader@nemours.org (M.W.S.); lydia.fisher@nemours.org (L.F.); william.mackenzie@nemours.org (W.G.M.); stuart.mackenzie@nemours.org (W.G.S.M.)

**Keywords:** Morquio syndrome, mucopolysaccharidosis IV, skeletal abnormality, gait patterns, skeletal dysplasia, children

## Abstract

Morquio syndrome (mucopolysaccharidosis IV/MPS IV) is a genetic disorder leading to skeletal abnormalities and gait deviations. Research on the gait patterns and lower extremity physical characteristics associated with skeletal dysplasia in children with MPS IV is currently limited. This research aimed to provide baseline gait patterns and lower limb skeletal alignment of children with MPS IV utilizing three-dimensional instrumented gait analysis. This Institutional Review Board-approved retrospective study evaluates the kinematics of the lower extremities of children with MPS IV during gait, comparing them with an age-matched group of typically developing children. Thirty-three children with MPS IV were included (8.6 ± 4.0 years old). Children with MPS IV walk with increased anterior pelvic tilt, knee valgus, knee flexion, external tibial torsion, and reduced walking speed and stride length (*p* < 0.001). Multiplanar abnormal alignment results in abnormal knee moments (*p* < 0.001). Limited correlations exist (*r* = 0.69–0.28) between dynamic three-dimensional measurements of knee varus/valgus and rotational alignment and traditional static two-dimensional measures (physical examination or radiographs) suggesting the possibility of knee instability during gait and the benefits of dynamic assessment.

## 1. Introduction

Mucopolysaccharidosis IV (MPS IV), or Morquio syndrome, is a rare progressive genetic disorder that has a well-known effect on lysosomal storage metabolism, which affects the development of connective tissue [1,2,3,4]. Signs and symptoms of MPS IV generally present within the first couple years of life and are progressive [2,5,6]. Common symptoms of MPS IV include skeletal manifestations affecting the spine and extremity alignment, decreased joint stability, and altered gait kinematics with wide-ranging levels of severity dependent on each child [2,3,4]. The incidence of MPS IV ranges from 1/76,000 births in Northern Ireland to 1/640,000 births in Australia, with some symptoms presenting as late as 3 years of age [1,4]. Most previous orthopedic studies on MPS IV have focused on the atlantoaxial stability of the cervical spine [2,7,8,9,10]; however, fewer studies have investigated the severity of lower extremity deformities including genu valgum, ankle valgus, progressive hip dysplasia, and other physical attributes, such as joint laxity, and their impact on 3D gait patterns [7,8]. Thus, further investigations to understand the variability in lower extremity deformities in children with MPS IV and the relationship between their severity and dynamic outcomes are warranted.

Early symptoms in children with MPS IV include short stature [3,5,11] and lower limb skeletal deformities, which cause impaired gait patterns such as reduced cadence, stride length, walking speed, and abnormal joint kinematics and kinetics [8]. Lower extremity orthopedic deformity of children with MPS IV is usually managed with surgical interventions aimed at improving progressive limb malalignment and gait, as well as minimizing functional limitations [2,6]. One of the most common targets of surgical intervention is genu valgum, where deformity correction can be attempted with guided growth utilizing hemiepiphysiodesis performed prior to skeletal maturity [6].

While lower extremity radiographs are typically used for the evaluation of deformities in children with MPS IV as part of the preoperative planning, they do not provide information about the dynamic function of the patient [12]. Three-dimensional (3D) instrumented gait analysis (IGA) may be used in addition to radiographs to provide the dynamic motion during preoperative planning; IGA has already been shown to aid in the decision-making of other conditions [13]. There is limited literature on the evaluation of the lower extremity utilizing 3D IGA for children with MPS IV. Previous research in a small sample population at our institution reported that children with MPS IV had a consistently abnormal gait pattern with reduced cadence and stride length, slower walking speeds, and multiplanar lower extremity boney malalignment when compared with typically developing children [8]. The objective of this work was to expand on the description of deviations in lower limb 3D gait patterns, physical examination (PE), and radiographic outcomes on a larger population of children with MPS IV. Additionally, dynamic 3D (IGA) and static two-dimensional (PE and radiographs) outcomes in the coronal and transverse plane will be compared to determine the potential benefits of complementing measurement techniques to improve treatment guidance in children with MPS IV.

## 2. Materials and Methods

Approval for this retrospective study was granted by our Institutional Review Board. Inclusion criteria consisted of confirmed diagnosis of Morquio syndrome, IGA prior to any lower extremity surgery, and under the age of 18 years at the time of gait analysis. 

Three-dimensional kinematic and kinetic patterns of children with MPS IV were collected utilizing a 12-camera motion capture system (Qualisys, Göteborg, Sweden) and AMTI force plates (Advanced Mechanical Technology, Inc., Watertown, MA, USA). Experienced physical therapists placed 34 reflective markers on participants based on the Cleveland marker set convention [14]. Children walked across the gait laboratory at a self-selected pace along a 12-m walkway at least six times to ensure that sufficient gait cycles for each limb were recorded. Noteworthy outcome variables for the IGA included the following measurements: cadence, forward velocity, stride length, and 3D pelvis, hip, knee, and ankle kinematics and kinetics. Forward velocity and stride length were further normalized based on participant’s height to account for height differences between children with MPS IV and those with typical development.

Children with MPS IV also underwent a PE performed by a physical therapist to evaluate the passive motion of each joint. Three-dimensional IGA data from children with MPS IV were compared with an age-matched cohort of typically developing children from our clinical database using the same data capture protocol as per our gait analysis laboratory standards of practice. Relevant PE hip, knee, and ankle values for typically developing children were obtained from the literature [15]. Furthermore, available anteroposterior radiographic image measurements of knee varus/valgus were retrieved from patients’ charts and compared with published norms [16]. All IGA and PE for each child were conducted at our institution in our gait analysis laboratory.

### Statistics

Two-tailed independent sample t-tests with unequal variance were utilized to compare children with MPS IV with the cohort of typically developing children for temporal/spatial, kinematic, and kinetic results collected during walking; a two-tailed independent sample t-test from the mean, standard deviation and sample size formula was calculated for PE and radiographic measurements using values from the literature [15,16]. A further independent t-test was used to compare normalized velocity, and knee coronal and transverse measurements for 3D IGA, PE, and radiographic data between male and female participants. 

Pearson’s correlation coefficients for knee measurements between 3D IGA and PE, 3D IGA and radiograph, and PE and radiograph in the coronal plane and between 3D IGA and PE in the transverse plane were calculated to determine whether there was a relationship between these measurements in this population. To investigate whether knee deformities in the coronal and transverse plane were related to age, further regression analyses between age and PE, and age and 3D IGA were calculated.

## 3. Results

Forty-seven children with MPS IV were identified from the retrospective database search. Of these children, 16 were excluded as they had lower limb surgery prior to their first gait analysis, or they were older than 18 years at the time of their gait analysis. Thus, 31 children with MPS IV (62 limbs), were included in the analysis (average age 8.6 ± 4.0 years; 18 female). 

Children with MPS IV had significant gait deviations from their typically developed peers (Table 1). Children with MPS IV tended to walk with limited raw and normalized stride length (*p* < 0.0001 and *p* = 0.0172, respectively), raw and normalized velocity (*p* < 0.0001 and *p* = 0.0053, respectively), and cadence (*p* = 0.0087). There was excessive lumbar lordosis with anterior trunk lean, anterior pelvic tilt, and resulting limited hip extension (*p* < 0.001). Knees demonstrated knee valgus and external tibial torsion (*p* < 0.001) with a large amount of variation within the group (knee valgus 38° to −2°; external tibial torsion 55° to −4°), resulting in a significantly abnormal knee varus moment (*p* < 0.001). Knees were excessively flexed during stance (*p* < 0.001).

Table 2 shows the mean, standard deviation, and passive range of motion measurements of the lower limb joints recorded during the physical examination by a physical therapist. Physical examination values show significant external tibial torsion and knee valgus (*p* < 0.001).

Typically developed knee varus-valgus and transmalleolar axis values were obtained from the literature for PE (53 children 4–16 years of age) [17] and radiographic measurements (14 children 8–9 years of age) [15]. PE, physical examination.

There was a strong correlation between PE and 3D gait measurements for knee deviations in the coronal plane (*r* = 0.68, *p* < 0.0001), and a weak correlation in the transverse plane (*r* = 0.33, *p* = 0.009; Table 3). There was a strong correlation between radiographic measurements and 3D gait values (*r* = 0.75, *p* < 0.0001), and a strong correlation between PE and radiographic measurements (*r* = 0.74, *p* < 0.0001). Age had a weak but significant correlation with increased transmalleolar axis (*r* = 0.3, *p* = 0.017).

## 4. Discussion

Children with a diagnosis of MPS IV present with short stature, progressive skeletal dysplasia, bone deformities, and joint and cervical spine instability, which result in an overall impaired function and reduced quality of life [3]. Routine clinical management of children with this condition involves PE for the quantification of physical abnormalities including growth rate, hip range of motion, and genu valgum and abnormal gait patterns, which are evident from as early as 2 years of age [11]. 

There is currently limited data in the literature as to the magnitude of gait deviation and lower limb bone and joint deformity in children with MPS IV in relation to typically developing children. While a previous paper from our group described 3D gait analysis, clinical examination, and radiographic data on 9 children diagnosed with MPS IV, this is the first study to report in a large number of patients, providing a further comparison on torsional measurements and correlations between 3D kinematics and physical examination. Results of this study demonstrate that children with Morquio syndrome walk with reduced walking speed, cadence, and stride length as well as altered kinematics and kinetics at every level, most prominently at the knee joint. While previous research has reported similar results [8], the number of subjects included was limited (nine children; 18 limbs). Multiplanar malalignment is often present but is variable in its presentation in this population, demonstrating the need for careful 3D assessment. The average child with MPS IV presented with knee valgus and external tibial torsion, but a closer look at the range of children studied showed variations from severe genu valgus and external tibial torsion to normal alignment in both planes. Small differences were noted between genders with males having slightly higher amounts of valgus. The noted differences may simply be due to the males and females in the study sample given the variation in severity of knee valgus in this population rather than a true effect of gender, but future work will need to clarify this further.

While the level of severity of the symptoms associated with MPS IV is unique to each patient with MPS IV [4], children with this rare genetic disorder show a decline in performing activities of daily living at around 10 years of age. Furthermore, there is evidence in the literature that factors such as surgical intervention and therapeutic treatment can positively impact activities of daily living in individuals with this condition [2]. For instance, Dhawale et al. reported 65% of surgically treated patients walked without assistance at greater than 6 year follow-up [7], suggesting improvement over the natural history reported by Montaño et al. [18]. Further longitudinal studies are necessary to monitor changes due to natural progression, and/or surgical treatment, and to determine the best practice to improve outcomes, depending on the individual’s baseline assessments.

Regression analysis between PE, 3D kinematics, and radiographs of the knees in the sagittal and coronal plane showed that, while these different techniques are correlated to a certain extent, they indeed complement each other, as they are obtained under different conditions and together provide a detailed holistic perspective of the anatomical structure and function of the lower limbs. Furthermore, both 3D motion analysis and PE data provide information in the transverse plane that standard anteroposterior and lateral plane imaging cannot. Indeed, 3D motion analysis data are of particular importance as they also offer dynamic information that neither radiographs nor PE are able to quantify.

Three-dimensional motion capture data are routinely used to help guide surgical management and evaluate outcomes in pediatric populations with the diagnosis of cerebral palsy, congenital talipes equinovarus, and arthrogryposis [19,20,21,22,23,24]. Similarly, this technology can also be used to measure gait data in children with MPS IV and assist the decision-making process for treatment management. Furthermore, 3D motion capture data can be used in conjunction with imaging and PE to objectively evaluate natural progression and post-surgical progress, and potentially recommend further treatment and/or rehabilitation protocols. While the number of individuals in our study was considerably greater than in previous publications, future research studies should attempt to recruit an even greater number of participants due to the relatively heterogeneous presentation of this condition.

### Limitations

Given the retrospective data collection from children with MPS IV from clinical evaluations at a specialized tertiary pediatric facility, these results may not be representative of all children with MPS IV. For instance, there is a large variability in phenotype with some children’s physical characteristics closely resembling those of typically developing children. As such, most of the children included in this study had their gait analysis session in preparation for imminent lower limb surgical procedures, indicating their lower limb deformities may have been at the severe end of the spectrum.

## 5. Conclusions

This study provides quantitative information on the atypical physical characteristics and dynamic gait patterns in children with MPS IV. Most evidently, children with this condition present with multiplanar lower extremity boney malalignment, most commonly with excessive knee valgus and external tibial torsion, though variation is present between patients. Clinical care of this complex population could benefit from the detailed analysis provided by PE and 3D motion capture for a clear understanding of their multilevel, multiplanar lower extremity malalignment in addition to the traditional two-dimensional methods of assessment (radiographs) that are typically used for preoperative planning.

## Figures and Tables

**Table 1:** The mean, standard deviation (SD), and range of temporal/spatial, kinematic, kinetic, and gait deviation index (GDI) values ^1^ [14] of children with Morquio syndrome (MPS IV) compared with a cohort of typically developed children.

Kinematic Variables	*n*	MPS IV							Typically Developed			
		Mean		SD	Min.		Max.		Mean		SD	*p*
Forward velocity (cm/s)	31	77.29		25.46	10.26		121.43		116.37		10.55	<0.0001
Normalized forward velocity	31	0.77		0.25	0.13		1.26		0.91		0.08	0.0053
Cadence (steps/min.)	31	123.49		24.37	58.80		162.36		137.09		12.96	0.0087
Stride length average (cm)	31	73.35		17.50	19.47		112.65		103.46		18.12	<0.0001
Normalized stride length	31	0.73		0.15	0.26		0.98		0.80		0.03	0.0172
Trunk forward tilt (°) ^2^	62	6	ant	13	5.94	post	65	ant	1.85	ant	1.4	0.0067
Pelvis forward tilt (°) ^2^	62	18	ant	5	9.73	ant	34	ant	12.98	ant	3	<0.0001
Hip flexion angle (°) ^3^	62	12	flex	17	13.91	ext	68	flex	4.29	ext	3	<0.0001
Hip adduction angle (°) ^4^	62	14	add	9	11.45	abd	30	add	8.86	add	2	<0.0001
Hip rotation angle (°) ^2^	62	3	extr	12	0.00	int	19	int	5.80	extr	4	0.1009
Knee flexion angle (°) ^2^	62	31	flex	18	0.99	ext	93	flex	18.13	flex	2	<0.0001
Knee flexion angle (°) ^5^	62	21	flex	14	6.32	ext	68	flex	7.72	flex	3	<0.0001
Knee valgus angle (°) ^2^	62	17	val	11	37.77	val	2	var	4.36	val	3	<0.0001
Knee valgus angle (°) ^3^	62	12	val	9	31.76	val	6	var	0.91	val	3	<0.0001
Knee valgus angle (°) ^4^	62	20	val	11	41.57	val	1	val	8.65	val	3	<0.0001
Tibial rotation angle (°) ^2^	62	27	extr	13	54.86	extr	4	int	3.10	extr	4	<0.0001
Foot orientation (°) ^2^	62	12	extr	14	51.18	extr	11	int	7.36	extr	2	0.0104
Peak knee abduction moment (nm/kg) ^6^	46	0.35	var	0.32	0.90	var	0.75	val	0.24	val	0	<0.0001
Foot varus-valgus position	62	10.70	var	41.76	97.00	var	97.50	val	30.00	var	0.00	0.0006
GDI average	62	71.27		18.95	20.52		102.38		100.00	^1^	10.0^1^	<0.001

^1^ Normative data obtained from the literature [14], ^2^ average during stance, ^3^ minimum during stance, ^4^ maximum during stance, ^5^ average at initial contact, ^6^ loading response. abd, abduction; add, adduction; ant, anterior; dflex, dorsiflexion; ext, extension; extr, external; flex, flexion; int, internal; post, posterior; val, valgus; var, varus.

**Table 2:** The mean, standard deviation (SD), and passive range of motion of children with Morquio syndrome.

Passive Range of Motion/Physical Examination	Morquio Syndrome					Typically Developing			
	*n*	Mean	SD	Max.	Min.	*n*	Mean	SD	*p*
Hip extension ^1^	62	−3.00	8.64	15	−20	53	12.5	5.4	<0.001
Hip abduction ^1^	62	37.55	8.83	50	10	53	37.7	6.9	0.918
Hip internal rotation ^1^	62	67.00	15.50	95	33	53	57.3	12.3	<0.001
Hip external rotation ^1^	62	32.52	16.14	90	−5	53	48.1	10.8	<0.001
Knee extension ^1^	62	−6.05	11.20	11	−40	53	4	5	<0.001
Popliteal angle (low) ^1^	62	23.37	13.18	55	0	53	25.6	10.5	0.315
Popliteal angle (high) ^1^	53	22.45	13.34	60	0	53	22.7	10.3	0.915
Knee valgus PE^1^	62	18.61	8.69	40	5	53	5	3	<0.001
Knee varus/valgus radiographs (varus−, valgus+) ^2^	56	23.38	10.74	50	1	14	6	1.9	<0.001
Transmalleolar axis PE ^1^	62	26.10	9.49	45	−5	53	16	5.9	<0.001
Dorsiflexion (knee flexed) ^1^	62	17.47	8.99	38	−17	53	26.9	6.6	<0.001
Dorsiflexion (knee extended) ^1^	62	11.21	9.22	30	−35	53	21.3	5.4	<0.001

Normative data obtained from the literature ^1^ [17] ^2^ [15].

**Table 3:** Physical examination (PE) vs. radiographic vs. three-dimensional (3D) gait analysis Pearson’s correlation.

	*r*	*r* ^2^	*p*
Knee varus/valgus			
3D kinematics vs. PE	0.68	0.46	<0.0001
PE vs. radiographic	0.74	0.55	<0.0001
3D kinematics vs. radiographic	0.75	0.56	<0.0001
TMA vs. tibial rotation			
3D kinematics vs. PE	0.33	0.11	0.01

Coronal and transverse plane knee correlations between 3D instrumented gait analysis and physical examination, 3D instrumented gait analysis and radiographic, and physical examination and radiographic measurements. TMA, transmalleolar axis. When data were compared by gender, the only significant differences noted were more valgus knee angles in stance (mean difference of 9°) and more knee valgus according to radiographs in males (mean difference of 9°) (*p* < 0.01).

## Data Availability

Data available on request due to restrictions, e.g., privacy or ethical. The data presented in this study are available on request from the corresponding author. The data are not publicly available due to patient confidentiality.
